# Brazilian Dialysis Survey from 1999 to 2023: trends in demographics, nutritional status, primary disease, and viral serology

**DOI:** 10.1590/2175-8239-JBN-2025-0036en

**Published:** 2025-06-20

**Authors:** Fabiana B. Nerbass, Helbert do N. Lima, Ricardo C. Sesso, Jocemir R. Lugon

**Affiliations:** 1 Fundação Pró-Rim, Joinville, SC, Brazil.; 2 Universidade da Região de Joinville, Joinville, SC, Brazil.; 3 Universidade Federal de São Paulo, São Paulo, SP, Brazil.; 4 Universidade Federal Fluminense, Niterói, RJ, Brazil.

**Keywords:** Renal Dialysis, Peritoneal Dialysis, Epidemiology, Renal Insufficiency, Chronic

## Abstract

**Introduction:**

Since 1999, the annual Brazilian Dialysis Survey (BDS) has served as one of the essential primary data sources on the chronic dialysis population in Brazil. We aimed to analyze and report trends in demographic and clinical characteristics and the prevalence of positive viral serology among chronic dialysis patients from 1999 to 2023.

**Methods:**

We compared trends in sex distribution, age (elderly >65 years), primary causes of chronic kidney disease (CKD), nutritional status, and the prevalence of positive serology for hepatitis B, hepatitis C, and HIV. The Mann-Kendall test was used to evaluate trends across the entire study period, as well as in the first and second halves.

**Results:**

The prevalence of men on dialysis therapy increased slightly from 57% to 59%, while the proportion of elderly individuals rose from 24.9% in 2006 to 36.7% in 2023. Significant changes were observed in nutritional status, with prevalence changes in all categories. Hypertension remained the most prevalent primary cause of CKD; however, its prevalence declined over time, while that of diabetes increased and that of glomerulonephritis decreased. The prevalence of positive serology for hepatitis B and C virus has decreased substantially, whereas that of HIV increased.

**Conclusion:**

Overtime, the most striking changes occurred in age, nutritional status, and viral serologies. During the analyzed period, the dialysis population in Brazil has become older and with a higher prevalence of overweight. On the other hand, the prevalence of positive serology for hepatitis B and mainly hepatitis C exhibited a dramatic fall.

## Introduction

Since 1999, the Brazilian Society of Nephrology (BSN) has been carrying out the Brazilian Dialysis Survey (BDS), a nationwide initiative aimed at collecting and analyzing epidemiological and clinical data from patients undergoing chronic dialysis. Over 24 years of data collection, the number of individuals on chronic dialysis has more than tripled. While some patient characteristics have remained stable, others have changed significantly.

In the present study, we examine and discuss key demographic characteristics—including sex, age, primary kidney disease, nutritional status, and prevalence of positive serology for hepatitis B, hepatitis C, and HIV.

## Methods

From 1999 to 2005, all dialysis centers registered with the BSN were contacted by the BSN office via direct phone calls. From 1999 and 2004 a mean of 534 centers participated. Participation rate was available only for 2005 (83.4%).

From 2006 to 2023, registered centers were invited to participate via email and, in later years, also through the BSN media channels. Participation in the survey was voluntary, with responding dialysis centers completing an online questionnaire hosted on the BSN website. On average, 46% (n = 329) of the contacted dialysis centers responded.

Data were reported in aggregated groups rather than at the individual level, including the prevalence of predetermined characteristics. For instance, managers of the centers were asked: what was the number of patients on dialysis on July 1^st^.? How many patients were males? How many are older than 65 years? etc. The population was mostly on hemodialysis. On average, the prevalence of patients on peritoneal dialysis was 8.7%.

All available data were included in the analysis, and the years of data collection varied across the studied variables, as detailed in Table 1.

**Table 1:** Summary of data collection frequency for variables in the Brazilian Dialysis Survey

Variable	First year of collection	Years not collected
Virus serology	1999	None
Proportion of those > 65 years	2006	2008, 2009, 2022
Primary kidney disease	2008	None
Sex	2009	2022
Nutritional status	2014	2020, 2021, 2022

### Statistical Analysis

The Mann-Kendall test was employed to evaluate monotonic trends in all variables over the whole study period as well as within the first and second halves of the timeline (e.g., 1999–2023, 1999–2009, and 2010–2023, respectively). An exception was made for nutritional status as data was only available for seven years. The strength and direction of the trends were quantified using Kendall’s Tau (τ), a rank correlation coefficient ranging from −1 to +1, where values close to −1 and +1 indicate strong negative and positive trends, respectively. The level of significance was set at p < 0.05. Statistical analyses were conducted using SPSS Statistics for Windows, version 21.0, and Python programming language, version 3.13.1.

## Results

**Figure 1:**
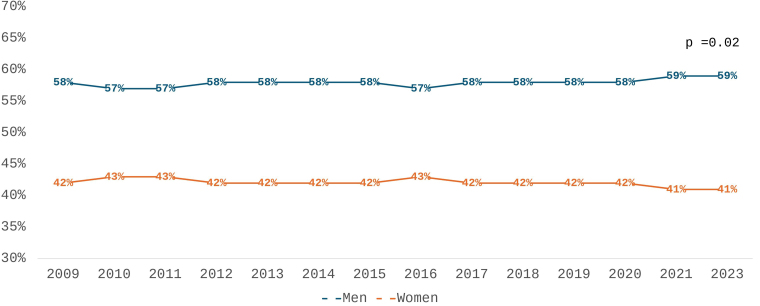
Distribution of men and women in chronic dialysis from 2009 to 2023.

Sex prevalence is presented in Table 2 and Figure 1. Over the 14-year follow-up, the proportion of male patients ranged from 57% to 59%, while female patients accounted for 41% to 43%. A moderate trend was observed across the entire period (τ = 0.41, p = 0.019), with a stronger trend emerging in the second half (2016–2023; τ = 0.67, p = 0.027).

**Table 2:** Prevalence of all variables over the follow-up time and results of the Mann-Kendall test

			Mann-Kendall test					
	Median		Whole period		1^st^ half		2^nd^ half	
Variables	1^st^ half	2^nd^ half	τ	p	τ	p	τ	p
Sex (men)	58%	58%	0.41	0.019	0.29	0.333	0.67	0.027
Age (≥ 65 years)	31%	35%	0.88	<0.001	0.79	0.008	0.79	0.007
Nutritional status*								
Underweight	9%		−0.86	0.008				
Normal	51%		−0.81	0.014				
Overweight	27%		0.81	0.014				
Obesity	13%		0.81	0.011				
CKD primary cause								
Hypertension	35%	34%	−0.45	0.012	−0.25	0.324	−0.11	0.789
Diabetes	28%	31%	0.74	<0.001	0.71	0.015	0.21	0.499
Glomerulonephritis	12.5%	9%	−0.84	<0.001	−0.64	0.026	−0.68	0.016
Polycystic kidney	4%	4%	−0.12	0.129	**	**	−0.25	0.19
Other/unknown	21%	23%	0.24	0.197	−0.79	0.006	0.43	0.167
Viral serology								
Hepatitis C	10.4%	3.2%	−0.99	<0.001	−0.97	<0.001	−0.98	<0.001
Hepatitis B	1.8%	0.8%	−0.80	<0.001	−0.80	<0.001	−0.61	0.006
HIV	0.5%	0.9%	0.80	<0.001	0.82	<0.001	0.77	<0.001

Notes –

*Results for the whole period due to the availability of data for only seven years;

**Statistics were computed because the variable is a constant.

**Figure 2:**
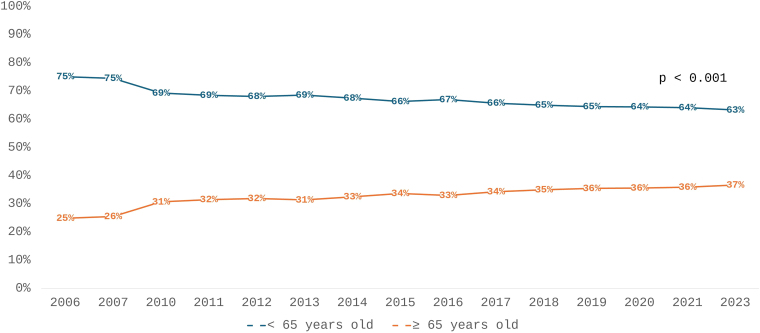
Prevalence of patients on chronic dialysis according to age (cut-off 65 years old) from 2006 and 2023.

The prevalence of patients aged 65 years or older steadily increased over time (Table 2 and Figure 2). In 2006, this age group comprised 25% of the chronic dialysis population, rising to 33% a decade later and reaching 36.7% by 2023. Significant trends were evident throughout the entire follow-up (τ = 0.88, p < 0.001) as well as in both halves of the period.

**Figure 3:**
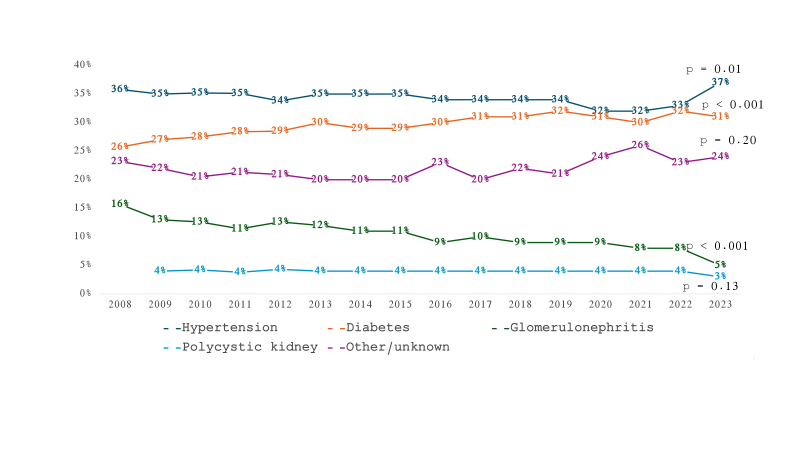
Distribution of dialysis patients according to chronic kidney disease primary cause from 2008 to 2023.

Changes in nutritional status classifications over time are detailed in Table 2 and Figure 3. There was a marked decline in the prevalence of underweight (τ = -0.85) and normal weight (τ = -0.81), alongside significant increases in overweight and obesity prevalence (τ = 0.81 for both).

**Figure 4:**
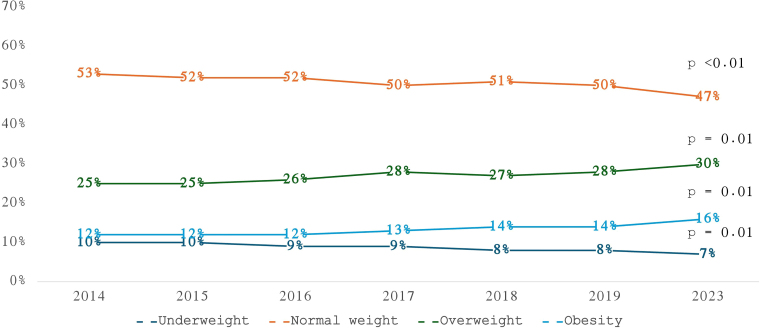
Distribution of patients according to nutritional status by body mass index from 2014 to 2023.

Trends in CKD primary cause are presented in Table 2 and Figure 4. Hypertension remained the leading cause of CKD throughout the study period, with a decreasing trend observed only when the entire period was considered (τ = -0.45). Diabetes prevalence increased significantly over the whole period (τ = 0.73), especially in the first half. In contrast, glomerulonephritis showed a strong decline (τ = -0.84), which persisted even when the follow-up time was split. Polycystic kidney disease prevalence remained stable, while other or unknown causes exhibited significant decreasing trends only during the first half of the follow-up.

**Figure 5:**
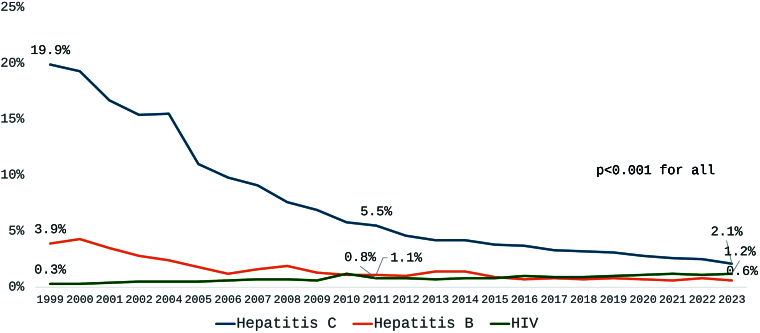
Prevalence of patients with positive serology for hepatitis B and C and HIV from 1999 to 2023.

Positive serology trends are outlined in Table 2 and Figure 5. Hepatitis C and B showed a very strong and significant decline in prevalence (τ = -0.99 and -0.80, respectively), while HIV prevalence exhibited a significant upward trend (τ = 0.80). These trends were statistically significant in both halves of the study period.

## Discussion

In this retrospective analysis of the primary BSN dataset on chronic dialysis patients in our country, we observed an increase in the age of this population, along with a sharp decline in the prevalence of positive serology for hepatitis C and B.

Sex distribution changed slightly over the 14 years of follow-up, with an increase in the proportion of males from 57% in 2009 to 59% in recent years. The absolute predominance of males among chronic dialysis patients is a global phenomenon, as is the observed gradual increase in this trend^1,2,3^. The reasons for these observations are currently unclear. Biological, cultural, and behavioral factors, along with their complex interactions, have been proposed to explain the persistent sex disparities observed worldwide, including unequal access to renal replacement therapy, and faster progression of CKD in men than in women^4,5^.

The steady rise in the proportion of elderly individuals undergoing dialysis, which increased from 25% to nearly 37% over the 17 years of follow-up, may be a reflection of the longer life expectancy of the Brazilian population^6^. While the total population grew by 8% between 2006 and 2022, the prevalence of individuals aged 65 years or older rose by 68%, representing 10.9% of the total population^7^.

Over the past decade, the most notable changes in the nutritional status of dialysis patients were a 30% reduction in the prevalence of underweight individuals (from 10% to 7%) and a 33% increase in the prevalence of obesity (from 12% to 16%). These trends in nutritional status align with broader national patterns^8^. However, compared to the general Brazilian population in 2019, the proportion of underweight individuals on dialysis is higher (8% vs. 1.6%) as is the proportion of normal weight patients (50% vs. 37%), while the prevalence of overweight (28% vs. 37%) and obesity (14% vs. 26%) is lower^9^. It is well known that several factors influence the nutritional and metabolic status of dialysis patients, increasing the risk of weight loss and malnutrition^10^.

Hypertension has consistently been the most prevalent primary cause of CKD since the beginning of the BDS. The most notable changes over the 15 years of follow-up include a rise in the proportion of individuals with diabetes, particularly during the first half, and a steady decline in the prevalence of glomerulonephritis. The increasing prevalence of diabetes as the underlying cause of CKD is a global phenomenon observed across various dialysis populations^11^. A recent study examining the burden of CKD attributable to type 2 diabetes across 204 countries from 1990 to 2019 reported a 21.8% global increase in incidence, with the most significant rise being in males aged 60–79 years in regions with a medium Socio-Demographic Index. Notably, in South and Latin America, the incidence increased by 35.9%, highlighting regional disparities in the impact of diabetes as the primary cause of CKD^12^. The decline in the prevalence of glomerulonephritis, from an average of 12% in the first half of the study to 8% in the second half, can be partially attributed to the rising impact of diabetes and hypertension as leading causes of CKD, a trend also observed in other populations^13^. Another contributing factor may be the impact of the COVID pandemic, which reduced access to kidney biopsies—an essential procedure for establishing the diagnosis of glomerulonephritis in particular—and may have contributed to the increasing number of undetermined causes among patients with kidney failure. However, the variations in the causes of CKD found over the period need to be considered with caution, as late symptoms and the lack of uniform access to more accurate diagnostic methods in different regions of the country may influence the precise distribution of renal failure etiologies.

The decrease in hepatitis C prevalence from nearly 20% of the dialysis population to only 2.1% over the 24 years of follow-up is remarkable. A systematic review and meta-analysis of studies evaluating HCV infection rates among hemodialysis patients in Brazil, excluding BDS data, also demonstrated a substantial decline in HCV prevalence between 1992 and 2015. The average HCV prevalence was 34% (95% CI: 26–43%) for studies conducted before 2001 compared to 11% (95% CI: 8–15%) for studies conducted after 2001^14^. Globally, the prevalence of HCV infection among dialysis patients has also shown a declining trend attributed to the availability of erythropoietin-stimulating agents, reduction in transfusions, increased infection control measures, routine serological screening, and the introduction of direct-acting antivirals for HCV treatment^15,16^. In recent years, antivirus C drug therapy has been provided by the government at no cost to patients in Brazil.

A continuous decline was also observed for hepatitis B prevalence, although there is a lack of information in the recent scientific literature regarding this topic. In 2003, the Dialysis Outcomes and Practice Patterns Study reported an average HBV prevalence of 3% and a median of 1.9% across 308 HD units in seven developed countries^17^. This decline is largely attributed to the implementation of HBV immunization programs and the isolation of HBsAg-positive patients within HD units^18^.

In contrast, while still relatively low, the prevalence of patients with positive HIV serology has quadrupled, increasing from 0.3% to 1.2%. Data from the United States and Europe suggest that the prevalence of HIV among the end-stage renal disease (ESRD) population ranges between 0.5% and 1.5%^19^. Despite a decline in the overall detection rates of HIV in the Brazilian population over recent decades^20^, this increase can be partly attributed to the improved life expectancy of individuals living with HIV^21^. As this population ages, kidney disease has become an increasingly common comorbidity^19^.

As study limitations, we should mention the reliance on estimates derived from electronic data through voluntary participation, the use of aggregated data at the dialysis center level, and the lack of validation of responses.

In conclusion, this retrospective analysis provides valuable insights into the evolving characteristics of the chronic dialysis population in Brazil over more than two decades. The findings underscore the effects of demographic and epidemiological transitions, including an aging population with chronic diseases, a marked decline in the prevalence of hepatitis B and especially C, and changes in the nutritional status and in the cause of primary kidney disease. The observed trends reflect advancements in public health measures and broader societal changes.

## Data Availability

The data may be available from the corresponding author upon reasonable request and with appropriate institutional approvals.
