# Supplementary material for: Brazilian Dialysis Survey from 1999 to 2023: trends in demographics, nutritional status, primary disease, and viral serology
Source: J Bras Nefrol. 2025 Jun 20;47(3):e20250036. doi: 10.1590/2175-8239-JBN-2025-0036en (PMC12187089; doi:10.1590/2175-8239-JBN-2025-0036en)
Supplement: Translation

# Translation: Censo Brasileiro de Diálise de 1999 a 2023: tendências em demografia, estado nutricional, doença primária e sorologia viral

**DOI:** 10.1590/2175-8239-JBN-2025-0036pt

**Authors:** Fabiana B. Nerbass, Helbert do N. Lima, Ricardo C. Sesso, Jocemir R. Lugon

**Affiliations:** 1 Fundação Pró-Rim, Joinville, SC, Brasil.; 2 Universidade da Região de Joinville, Joinville, SC, Brasil.; 3 Universidade Federal de São Paulo, São Paulo, SP, Brasil.; 4 Universidade Federal Fluminense, Niterói, RJ, Brasil.

**Keywords:** Diálise Renal, Diálise Peritoneal, Epidemiologia, Insuficiência Renal Crônica

## Abstract

**Introdução:**

Desde 1999, o Censo Brasileiro de Diálise (CBD), realizado anualmente, tem sido uma das principais fontes primárias de dados sobre a população em diálise crônica no país. Nosso objetivo foi analisar e relatar tendências nas características demográficas e clínicas e a prevalência de sorologia viral positiva entre pacientes de diálise crônica de 1999–2023.

**Métodos:**

Comparamos tendências na distribuição por sexo, idade (idosos >65 anos), causas primárias de doença renal crônica (DRC), estado nutricional e prevalência de sorologia positiva para hepatite B, C e HIV. Utilizamos o teste de Mann-Kendall para avaliar as tendências durante todo o período do estudo, bem como na primeira e na segunda metades.

**Resultados:**

A prevalência de homens em terapia dialítica aumentou ligeiramente de 57% para 59%; a proporção de idosos aumentou de 24,9% em 2006 para 36,7% em 2023. Houve mudanças significativas no estado nutricional, com alterações de prevalência em todas as categorias. A hipertensão manteve-se como causa primária mais prevalente de DRC; entretanto, sua prevalência diminuiu ao longo do tempo, enquanto a do diabetes aumentou e da glomerulonefrite diminuiu. A prevalência de sorologia positiva para os vírus da hepatite B e C diminuiu substancialmente, enquanto a do HIV aumentou.

**Conclusão:**

Com o tempo, as alterações mais marcantes ocorreram em idade, estado nutricional e sorologias virais. Durante o período analisado, a população em diálise no Brasil tornou-se mais velha e com maior prevalência de sobrepeso. Por outro lado, a prevalência de sorologia positiva para hepatite B e principalmente para hepatite C apresentou queda drástica.

## Introdução

Desde 1999, a Sociedade Brasileira de Nefrologia (SBN) vem realizando o Censo Brasileiro de Diálise (CBD), uma iniciativa de âmbito nacional que visa coletar e analisar dados epidemiológicos e clínicos de pacientes submetidos à diálise crônica. Ao longo de 24 anos de coleta de dados, o número de indivíduos em diálise crônica mais do que triplicou. Embora algumas características dos pacientes tenham se mantido estáveis, outras apresentaram mudanças significativas.

No presente estudo, examinamos e discutimos as principais características demográficas–incluindo sexo, idade, causa primária da doença renal crônica (DRC), estado nutricional e prevalência de sorologia positiva para hepatite B, hepatite C e HIV.

## Métodos

De 1999 a 2005, todos os centros de diálise registra­dos na SBN foram contatados pelo escritório da entidade por meio de ligações telefônicas. De 1999 a 2004, uma média de 534 centros participaram. A taxa de participação foi disponibilizada apenas para 2005 (83,4%).

De 2006 a 2023, os centros registrados foram convidados a participar por e-mail e, nos últimos anos, também por meio dos canais de mídia da SBN. A participação no censo foi voluntária e os centros de diálise respondentes preencheram um questionário online disponibilizado no site da SBN. Em média, 46% (n = 329) dos centros de diálise contatados enviaram respostas.

Os dados foram relatados em grupos agregados e não em nível individual, incluindo a prevalência de características predeterminadas. Por exemplo, perguntou-se aos gestores dos centros: qual era o número de pacientes em diálise em 1º de julho? Quantos pacientes eram do sexo masculino? Quantos têm mais de 65 anos? etc. A população foi composta majoritariamente por pacientes em hemodiálise. Em média, a prevalência de pacientes em diálise peritoneal foi de 8,7%.

Todos os dados disponíveis foram incluídos na análise, e os anos de coleta dos dados diferiram entre as variáveis estudadas, conforme detalhado na Tabela 1.

**Tabela 1:** Resumo da frequência de coleta de dados para as variáveis do Censo Brasileiro de Diálise

Variável	Primeiro ano de coleta	Anos sem coleta
Sorologia viral	1999	Nenhum
Proporção de pessoas > 65 anos	2006	2008, 2009, 2022
Causa primária da DRC	2008	Nenhum
Sexo	2009	2022
Estado nutricional	2014	2020, 2021, 2022

Abreviação – DRC: doença renal crônica.

### Análise Estatística

O teste de Mann-Kendall foi empregado para avaliar tendências monotônicas em todas as variáveis ao longo de todo o período do estudo, bem como na primeira e segunda metades do intervalo temporal (por exemplo, 1999–2023, 1999–2009 e 2010–2023, respectivamente). Com exceção para o estado nutricional, uma vez que os dados somente estavam disponíveis para sete anos. A força e a direção das tendências foram quantificadas por meio do Tau de Kendall (τ), um coeficiente de correlação que varia de −1 a +1, em que valores próximos a −1 e +1 indicam fortes tendências negativas e positivas, respectivamente. O nível de significância foi definido como p < 0,05. As análises estatísticas foram realizadas utilizando o programa SPSS Statistics for Windows, versão 21.0, e a linguagem de programação Python, versão 3.13.1.

## Resultados

A prevalência de sexo é apresentada na Tabela 2 e na Figura 1. Ao longo dos 14 anos de acompanha­mento, a proporção de pacientes do sexo masculino variou de 57% a 59%, enquanto os pacientes do sexo feminino representaram 41% a 43%. Uma tendência moderada foi observada durante todo o período (τ = 0,41; p = 0,019), com uma tendência mais forte surgindo na segunda metade (2016–2023; τ = 0,67; p = 0,027).

**Tabela 2:** Prevalência de todas as variáveis durante o período de acompanhamento e resultados do teste de Mann-Kendall

			Teste de Mann-Kendall					
	Mediana		Período total		1ª metade		2ª metade	
Variáveis	1ª metade	2ª metade	τ	p	τ	p	τ	p
Sexo (homens)	58%	58%	0.41	0.019	0.29	0.333	0.67	0.027
Idade (≥ 65 anos)	31%	35%	0,88	<0,001	0,79	0,008	0,79	0,007
Estado nutricional*								
Baixo peso	9%		−0,86	0,008				
Normal	51%		−0,81	0,014				
Sobrepeso	27%		0,81	0,014				
Obesidade	13%		0,81	0,011				
Causa primária da DRC								
Hipertensão	35%	34%	−0,45	0,012	−0,25	0,324	−0,11	0,789
Diabetes	28%	31%	0,74	<0,001	0,71	0,015	0,21	0,499
Glomerulonefrite	12,5%	9%	−0,84	<0,001	−0,64	0,026	−0,68	0,016
Rim policístico	4%	4%	−0,12	0,129	**	**	−0,25	0,19
Outras/desconhecidas	21%	23%	0,24	0,197	−0,79	0,006	0,43	0,167
Sorologia viral								
Hepatite C	10,4%	3,2%	−0,99	<0,001	−0,97	<0,001	−0,98	<0,001
Hepatite B	1,8%	0,8%	−0,80	<0,001	−0,80	<0,001	−0,61	0,006
HIV	0,5%	0,9%	0,80	<0,001	0,82	<0,001	0,77	<0,001

Notas –

*Resultados para o período total, devido à disponibilidade de dados para apenas sete anos;

**As estatísticas foram computadas porque a variável é uma constante.

**Figura 1:** Distribuição de homens e mulheres em diálise crônica no período de 2009 a 2023.

A prevalência de pacientes com 65 anos ou mais aumentou de forma constante ao longo do tempo (Tabela 2 e Figura 2). Em 2006, essa faixa etária compreendia 25% da população em diálise crônica, aumentando para 33% uma década depois e chegando a 36,7% em 2023. Tendências significativas foram evidentes durante todo o acompanhamento (τ = 0,88; p < 0,001), bem como nas duas metades do período.

**Figura 2:** Prevalência de pacientes em diálise crônica de acordo com a idade (ponto de corte de 65 anos) entre 2006 e 2023.

As mudanças nas classificações do estado nutricional ao longo do tempo estão detalhadas na Tabela 2 e na Figura 3. Houve um declínio acentuado na prevalência de baixo peso (τ = −0,85) e peso normal (τ = −0,81), juntamente com aumentos significativos nas prevalências de sobrepeso e obesidade (τ = 0,81 para ambos).

**Figura 3:** Distribuição de pacientes em diálise de acordo com a causa primária da doença renal crônica no período de 2008 a 2023.

As tendências na causa primária da DRC são apresentadas na Tabela 2 e na Figura 4. A hipertensão manteve-se como a principal causa de DRC durante todo o período do estudo, com uma tendência decrescente observada apenas quando considerado o período total (τ = −0,45). A prevalência de diabetes aumentou significativamente ao longo de todo o período (τ = 0,73), especialmente na primeira metade. Em contraste, a glomerulonefrite apresentou um acentuado declínio (τ = −0,84), que persistiu mesmo quando o período de acompanhamento foi dividido. A prevalência de doença renal policística permaneceu estável, enquanto causas classificadas como “outras” ou “desconhecidas” apresentaram tendências significativas de redução somente durante a primeira metade do acompanhamento.

**Figura 4:** Distribuição de pacientes de acordo com o estado nutricional por índice de massa corporal entre 2014 e 2023.

As tendências de sorologia positiva estão descritas na Tabela 2 e na Figura 5. As hepatites C e B apresentaram um declínio bastante acentuado e significativo em sua prevalência (τ = −0,99 e −0,80, respectivamente), enquanto a prevalência do HIV apresentou uma tendência significativa de aumento (τ = 0,80). Essas tendências foram estatisticamente significativas em ambas as metades do período de estudo.

**Figura 5:** Prevalência de pacientes com sorologia positiva para hepatite B e C e HIV no período de 1999 a 2023.

## Discussão

Nessa análise retrospectiva do principal conjunto de dados da SBN sobre pacientes em diálise crônica em nosso país, observamos um aumento na idade dessa população, juntamente com um declínio acentuado na prevalência de sorologia positiva para hepatite C e B.

A distribuição por sexo apresentou uma ligeira alteração ao longo dos 14 anos de acompanhamento, com um aumento na proporção de homens de 57% em 2009 para 59% nos últimos anos. A predominância absoluta do sexo masculino entre os pacientes de diálise crônica é um fenômeno mundial, assim como o aumento gradual observado nessa tendência^1-3^. As razões para essas observações permanecem, até o momento, pouco esclarecidas. Fatores biológicos, culturais e comportamentais, juntamente com suas complexas interações, têm sido propostos para explicar as persistentes disparidades entre os sexos observadas globalmente, incluindo o acesso desigual à terapia renal substitutiva e a progressão mais rápida da DRC em homens do que em mulheres^4,5^.

O aumento constante na proporção de idosos submetidos à diálise, que aumentou de 25% para quase 37% ao longo dos 17 anos de acompanhamento, reflete a maior expectativa de vida da população brasileira^6^. Embora a população total tenha crescido 8% entre 2006 e 2022, a prevalência de indivíduos com 65 anos ou mais aumentou 68%, representando 10,9% da população total^7^.

Na última década, as mudanças mais notáveis no estado nutricional dos pacientes em diálise foram uma redução de 30% na prevalência de indivíduos com baixo peso (de 10% para 7%) e um aumento de 33% na prevalência de obesidade (de 12% para 16%). Essas tendências no estado nutricional estão alinhadas com padrões nacionais^8^. No entanto, em comparação com a população brasileira geral em 2019, a proporção de indivíduos com baixo peso em diálise é maior (8% vs. 1,6%), assim como a proporção de pacientes com peso normal (50% vs. 37%), enquanto as prevalências de sobrepeso (28% vs. 37%) e obesidade (14% vs. 26%) são mais baixas^9^. É sabido que diversos fatores influenciam o estado nutricional e metabólico dos pacientes em diálise, aumentando o risco de perda de peso e desnutrição^10^.

A hipertensão tem sido, de forma consistente, a causa primária mais prevalente de DRC desde o início do CBD. As mudanças mais notáveis ao longo dos 15 anos de acompanhamento incluem um aumento na proporção de indivíduos com diabetes, especialmente durante a primeira metade do período, e um declínio constante na prevalência de glomerulonefrite. O aumento da prevalência do diabetes como causa subjacente da DRC é um fenômeno global observado em diversas populações em terapia dialítica^11^. Um estudo recente que examinou o ônus da DRC atribuível ao diabetes tipo 2 em 204 países, entre 1990 e 2019, relatou um aumento global de 21,8% na incidência, sendo que o aumento mais significativo ocorreu em homens com idade entre 60 e 79 anos em regiões com Índice Sociodemográfico médio. Notavelmente, na América do Sul e na América Latina, a incidência aumentou em 35,9%, evidenciando as disparidades regionais no impacto do diabetes como a principal causa de DRC^12^. O declínio na prevalência de glomerulonefrite, de uma média de 12% na primeira metade do estudo para 8% na segunda metade, pode ser parcialmente atribuído ao impacto crescente do diabetes e da hipertensão como principais causas de DRC, uma tendência também observada em outras populações^13^. Outro fator contribuinte pode ter sido o impacto da pandemia da COVID, que reduziu o acesso a biópsias renais – um procedimento essencial para estabelecer o diagnóstico de glomerulonefrite em particular – e pode ter contribuído para o aumento do número de causas indeterminadas entre os pacientes. No entanto, as variações nas causas de DRC encontradas durante o período devem ser consideradas com cautela, uma vez que os sintomas tardios e a falta de acesso uniforme a métodos diagnósticos de maior acurácia em diferentes regiões do país podem influenciar a distribuição precisa das etiologias da DRC.

A redução na prevalência de hepatite C, de quase 20% da população em diálise para apenas 2,1% ao longo dos 24 anos de acompanhamento, é notável. Uma revisão sistemática e meta-análise de estudos que avaliaram as taxas de infecção pelo HCV entre pacientes em hemodiálise no Brasil, excluindo os dados do CBD, também demonstrou um declínio substancial na prevalência de HCV entre 1992 e 2015. A prevalência média de HCV foi de 34% (IC 95%: 26–43%) para estudos realizados antes de 2001, em comparação a 11% (IC 95%: 8–15%) para estudos realizados após 2001^14^. Mundialmente, a prevalência de infecção por HCV entre pacientes em diálise também apresentou uma tendência de queda, atribuída à disponibilidade de agentes estimuladores da eritropoiese, à redução no número de transfusões, ao aumento das medidas de controle de infecções, à triagem sorológica de rotina e à introdução de antivirais de ação direta para o tratamento do HCV^15,16^. Nos últimos anos, a terapia medicamentosa contra o vírus C tem sido disponibilizada de forma gratuita pelo governo para os pacientes no Brasil.

Também foi observado um declínio contínuo na prevalência de hepatite B, embora haja uma falta de informações na literatura científica recente sobre esse tema. Em 2003, o Dialysis Outcomes and Practice Patterns Study relatou uma prevalência média de HBV de 3% e uma mediana de 1,9% entre 308 unidades de HD em sete países desenvolvidos^17^. Essa redução é atribuída, em grande parte, à implementação de programas de imunização contra o HBV e ao isolamento de pacientes HBsAg-positivos nas unidades de HD^18^.

Em contrapartida, embora ainda relativamente baixa, a prevalência de pacientes com sorologia positiva para o HIV quadruplicou, aumentando de 0,3% para 1,2%. Dados provenientes dos Estados Unidos e da Europa sugerem que a prevalência do HIV entre a população com doença renal em estágio terminal varia entre 0,5% e 1,5%^19^. Apesar da redução nas taxas gerais de detecção do HIV na população brasileira nas últimas décadas^20^, esse aumento pode ser parcialmente atribuído à melhora na expectativa de vida dos indivíduos que convivem com o HIV^21^. À medida que essa população envelhece, a doença renal se torna uma comorbidade cada vez mais comum^19^.

Como limitações do estudo, devemos mencionar a dependência de estimativas derivadas de dados eletrônicos por meio de participação voluntária, o uso de dados agregados em nível de centro de diálise e a falta de validação das respostas.

Em conclusão, essa análise retrospectiva fornece informações valiosas sobre a evolução das características da população em diálise crônica no Brasil ao longo de mais de duas décadas. Os achados destacam os efeitos das transições demográficas e epidemiológicas, incluindo o envelhecimento da população com doenças crônicas, um declínio acentuado na prevalência da hepatite B e, principalmente, da C, e mudanças no estado nutricional e na causa primária de doenças renais. As tendências observadas refletem os avanços nas medidas de saúde pública e amplas mudanças sociais.

## Data Availability

Os dados podem ser disponibilizados pelo autor correspondente mediante solicitação razoável e com as devidas aprovações institucionais.
